# Stakeholders’ perceptions of policy options to support the integration of community health workers in health systems

**DOI:** 10.1186/s12960-019-0348-6

**Published:** 2019-02-18

**Authors:** Onyema Ajuebor, Giorgio Cometto, Mathieu Boniol, Elie A. Akl

**Affiliations:** 1 Health Workforce Department, World Health Organization, 20 Avenue Appia, CH-1211 Geneva 27, Switzerland; 2 Department of Internal Medicine, American University of Beirut, Beirut, Lebanon

**Keywords:** Community health workers, Health systems, Stakeholders, Health planning guidelines

## Abstract

**Background:**

Community health workers (CHWs) are an important component of the health workforce in many countries. The World Health Organization (WHO) has developed a guideline to support the integration of CHWs into health systems. This study assesses stakeholders’ valuation of outcomes of interest, acceptability and feasibility of policy options considered for the CHW guideline development.

**Methods:**

A cross-sectional mixed methods (quantitative and qualitative) study targeting stakeholders involved directly or indirectly in country implementation of CHW programmes was conducted in 2017. Data was collected from 96 stakeholders from five WHO regions using an online questionnaire. A Likert scale (1 to 9) was used to grade participants’ assessments of the outcomes of interest, and the acceptability and feasibility of policy options were considered.

**Results:**

All outcomes of interest were considered by at least 90% of participants as ‘important’ or ‘critical’. Most critical outcomes were ‘improved quality of CHW health services’ and ‘increased health service coverage’ (91.5% and 86.2% participants judging them as ‘critical’ respectively). Out of 40 policy options, 35 were considered as ‘definitely acceptable’ and 36 ‘definitely feasible’ by most participants. The least acceptable option (37% of participants rating ‘definitely not acceptable’) was the selection of candidates based on age. The least feasible option (29% of participants rating ‘definitely not feasible’) was the selection of CHWs with a minimum of secondary education.

**Conclusion:**

Outcomes of interest and policy options proposed were rated highly by most stakeholders. This finding helps to reinforce their usefulness in meeting the expectations of the CHW guideline end-users to properly integrate CHWs into health systems.

**Electronic supplementary material:**

The online version of this article (10.1186/s12960-019-0348-6) contains supplementary material, which is available to authorized users.

## Background

The term ‘community health workers’ refers to diverse types of health workers that deliver elementary health services in communities [1]. While there is no universally agreed definition of community health workers (CHWs), the International Labour Organization (ILO) defines them as workers who ‘… provide health education and referrals for a wide range of services, and provide support and assistance to communities, families and individuals with preventive health measures and gaining access to appropriate curative health and social services...’ [2]. CHWs serve as a link between providers of health, social and community services and communities that may have difficulty in accessing these services.

Societies have used CHWs for a long time with varying levels of popularity and success. One of the earliest known example dates to the 1920s when Chinese ‘Farmer Scholars’, later known as the ‘Barefoot Doctors’, were trained for 3 months to work in rural communities [3]. Despite the long history of CHW programmes operating in diverse climes, health systems often have neglected this group of health workers and have failed to recognize them formally, especially by excluding them from the roster of health workers legally allowed to deliver services in health systems. This, coupled with inadequate monitoring and evaluation mechanisms, has resulted in a reduced ability to quantify CHW contributions to health system performance [3–6].

In the 1970s, the World Health Organization (WHO) specifically recognized the contribution of CHWs to achieving primary health care (PHC) in countries. The Declaration of Alma-Ata states that ‘primary health care relies, at local and referral levels, on health workers, including physicians, nurses, midwives, auxiliaries and community workers as applicable, as well as traditional practitioners as needed, suitably trained socially and technically to work as a health team and to respond to the expressed health needs of the community’ [7]. The goal to enshrine integrated people-centred primary care through the concept of universal health coverage (UHC) is one that is still alive today. Consecutive biennial World health reports in 2006, 2008 and 2010 [8–10] recognized the challenges affecting all health workers and argued for scaling up the health workforce as a critical measure to expand health services coverage.

To support capacity building of health workforce in countries, WHO, member states and partners developed the Global Strategy on Human Resources for Health: Workforce 2030 [11, 12]. The strategy encourages countries to adopt a diverse, sustainable skills mix that harnesses the potential of community health workers and mid-level health workers in inter-professional primary care teams. The ultimate aim is to achieve the third Sustainable Development Goal (SDG 3) to ‘ensure healthy lives and promote wellbeing for all, at all ages’. The contributions of community health workers are further highlighted by the report of the United Nations High-Level Commission on Health Employment and Economic Growth, which underscores their effectiveness and advocates for their recognition and support [13].

Despite potential added value of CHWs to health systems, policy guidance supporting the strengthening and scale-up of CHW programmes generally has been lacking, especially in developing countries where the programmes are most needed. To help countries address this gap, WHO developed a guideline to optimize community health worker programmes [14, 15, 16]. WHO guidelines typically follow the GRADE methodology as indicated in the WHO Handbook for Guideline Development [17]. The WHO guideline development process begins with a scoping proposal headed by a steering group. Submission of the guidelines scoping to the WHO Guidelines Review Committee (GRC) then follows for review and approval, before the setup of the Guideline Development Group (GDG) and the External Review Group (ERG). The GDG consists of international multidisciplinary experts and subject leaders (including CHWs themselves in this case) and is responsible for formulating the population, intervention, control, outcome (PICO) questions and making recommendations based on the evidence obtained from systematic reviews and other evidence gathered for the guideline development. The role of the ERG is to review the synthesis of the evidence gathered and to submit the findings to the GDG who then decide on the final recommendations before the approval by the WHO GRC. The success of implementing guidelines could depend on its acceptance and feasibility by end-users including policy makers/planners, decision makers and other implementers. The WHO guideline development process therefore allows for the input of stakeholders to be taken into account in addition to findings from the systematic evidence syntheses. Recently conducted studies have noted the significance of stakeholders’ views in the implementation of health care programmes [18, 19].

This paper reports findings from the assessment of stakeholders’ valuation of CHW outcomes of interest, and the perceived acceptability and feasibility of the policy options considered for developing the CHW guideline recommendations. It aims at ensuring greater relevancy, inclusiveness and ownership of the recommendations at policy implementation and practice levels.

## Methods

This study was conducted using a cross-sectional mixed methods (quantitative and qualitative) design. It was conducted over a period of 6 months from February 2017 to July 2017. It targeted stakeholders, including community health workers, policy planners and government stakeholders involved directly or indirectly in country implementation of CHW programmes. The recruitment of respondents was done in a phased approach. The first selection of respondents was made among participants attending the 2017 Institutionalizing Community Health Conference held in Johannesburg, South Africa. Subsequently, respondents were identified from a wider audience including through the WHO Human Resources for Health contact list and the Health Information for All (HIFA) online platform to better target stakeholders from countries implementing CHW programmes. The WHO Human Resources for Health contact list and HIFA online forum enabled outreach to multisectoral groups including government representatives, health policy makers, academics, researchers, partner organizations, health professionals’ networks, publishers and librarians involved with CHWs. Participation was voluntary, and responses were made anonymous for privacy protection and to encourage openness, in line with WHO Research Ethics Review Committee (ERC) requirements.

### Survey design, data collection and analysis

The survey was designed to enable the collection of quantitative and qualitative data on CHW outcomes of interest, and acceptability and feasibility of policy options. A standard questionnaire (Additional file 1) was developed based on a previously published questionnaire used for the WHO health systems rehabilitation services guideline [18]. The questionnaire included the following characteristics of participants: highest academic degree attained, region represented, occupation, institution(s) represented, level of responsibility, gender and age. In addition, it included three sets of questions on (1) values assigned to the outcomes of relevance to the policy options under consideration and (2) acceptability and feasibility of the policy options under consideration. Policy options included in the study were selected from the intervention questions used for the development of the CHW guidelines. They are broadly categorized into three areas: (1) selection, education and certification; (2) management and supervision; and (3) integration in and support by health system and communities.

All questions on the profile characteristics of participants, apart from gender, were compulsory. The questions on occupation, institution and level of responsibility allowed for multiple entries. The policy options under consideration were identified from the draft WHO guideline planning proposal document and other CHW-related guidance documents as contained in the CHW guidelines development planning proposal. The outcomes were selected by the guideline panel during the first meeting of the GDG in October 2016. The guideline recommendations emerged from systematic reviews on the PICO framework and were complemented by implementation considerations and good practice recommendations. The WHO ERC provided the ethical clearance (ERC 0002869) for the study.

The survey was tested in a pilot study with three public health practitioners and WHO staff members to check for errors, ensure clarity and estimate the time required to complete the survey. The Survey Monkey platform was used to build the online questionnaire in English and in French.

All questions on outcomes of interest, acceptability and feasibility were labelled on Likert scales, coded from 1 to 9. Outcomes of interest where labelled as ‘not important’ for 1 to 3 values, ‘important’ for 4 to 6 values, and ‘critical’ for 7 to 9 values. With the same cutoff points, acceptability was labelled as ‘definitely not acceptable,’ ‘uncertain whether acceptable or not’ and ‘definitely acceptable’. Feasibility was labelled as ‘definitely not feasible’, ‘uncertain whether feasible or not’ and ‘definitely feasible’. In addition to the Likert scale answers, participants could provide qualitative assessments in the form of open comments.

Results from the Likert scale metrics were combined using the categories mentioned above and reported as percentages. Due to the nature of these categorical data, showing important skewness from visual inspection of their distribution and important departure from normality, no parametric statistical test was performed. A chi-square test therefore was used to evaluate discrepancies between distribution of answers in the feasibility versus acceptability of the policy options. To facilitate comparisons using one single value per component, Likert scale average score was also reported for each item. The qualitative data was analysed narratively and reported verbatim as relevant according to the policy options categorization:(1) selection, education and certification; (2) management and supervision; and (3) integration in and support by health system and communities. Data extraction and analysis were conducted using a subscription-based Survey Monkey platform and Microsoft Excel 2010.

## Results

### Profile of respondents

The figures below provide graphical illustrations of four of the seven respondent characteristics provided by the respondents to the study.

**Fig. 1:**
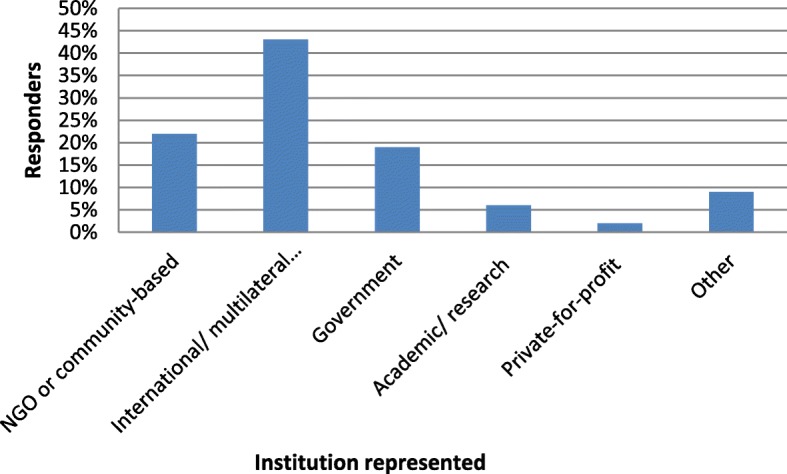
The percentage proportion of responders by the category of institution represented (*n* = 96 and multiple responses were allowed for this question)

**Fig. 2:**
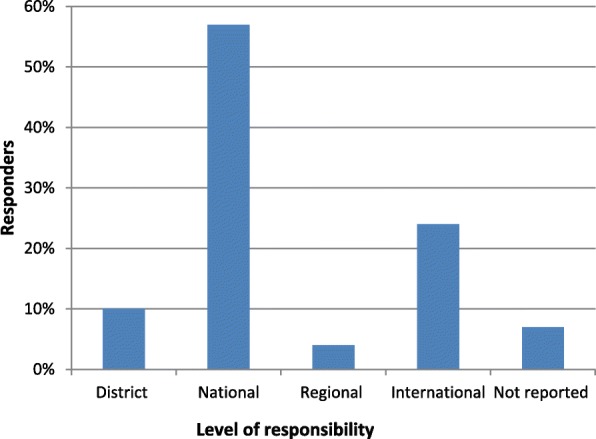
The percentage proportion of respondents according to their level of responsibility (*n* = 96 and multiple responses were allowed for this question)

Figure 1 summarizes the institutional representation of survey respondents. Approximately 70% of the respondents were working at district and national levels, with the remaining being mostly academics or researchers working at regional or international levels (Fig. 2).

**Fig. 3:**
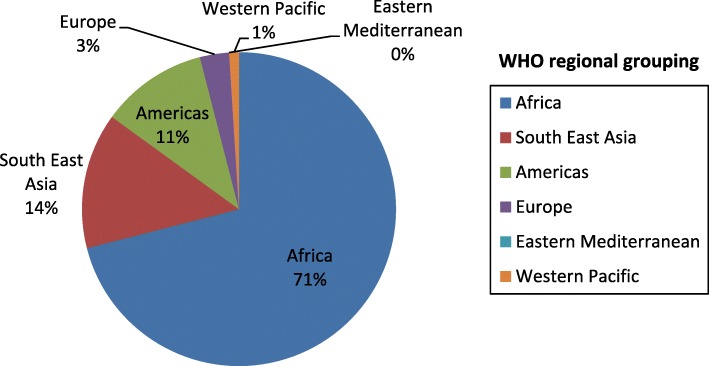
The percentage proportion of responders according to the WHO region they represent (*n* = 96)

More than 70% of the participants were representing the African region, where many CHW programmes are present and health services needs most apparent (Fig. 3).

**Fig. 4:**
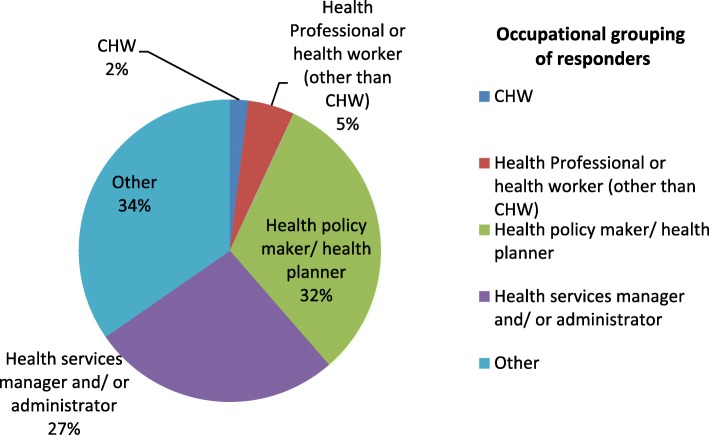
The percentage proportion of respondents according to their occupational grouping (*n* = 96 and multiple responses were allowed for this question)

More than 50% respondents were health policy makers, planners, health services managers or administrators. CHWs constituted only 2% of the respondents, although they were well represented in the Guidelines Development Group (Fig. 4).

### Values attached to the outcome measures

Table 1 summarizes the values assigned to the outcomes of relevance to the policy options under consideration. The categories of responses are grouped as follows: ‘not important’, ‘important’ and ‘critical’. More than 50% of the total responders across the three categories perceived all outcomes to be critical except the outcome related to *decrease in levels of discrimination* which was judged to be critical by 48.9% of the total respondents. The highest ratings among outcomes perceived as critical were for the following three outcomes: *quality of CHW health services*, *health services coverage* and *CHW competencies*. Among the outcomes identified as ‘important’, *decrease in levels of discrimination* was judged to be the highest, with 44.6% of the total responses. Outcomes following in the second and third place are *decreased CHW absenteeism* (39.1%) and *improved cost savings by patients* (34.78%). The following outcomes received the highest rating among those perceived by respondents to be ‘not important’: *improved family planning* (8.6%), *decrease in the levels of discrimination* (6.5%) and *decrease in preventable mortality rates* (5.3%).

**Table 1:** Values assigned to the outcomes of relevance for the policy options under consideration for the CHW guideline (*N* = 96)

Outcomes of relevance	Not important (%)	Important (%)	Critical (%)	Mean score from Likert scale
Increased CHW motivation	2.1	24.2	73.7	7.6
Improved CHW morale	1.1	31.6	67.4	7.3
Decreased CHW absenteeism	4.4	39.1	56.5	6.8
Increased CHW productivity	1.1	25.3	73.7	7.4
Improved CHW responsiveness	1.1	33.0	65.9	7.2
Decrease in CHW attrition rates	3.2	34.0	62.8	7.0
Improved CHW competencies	1.1	15.4	83.5	7.8
Increased access to care for patients	1.1	16.0	83.0	7.8
Increased health services coverage	0.0	13.8	86.2	8.0
Improved quality of CHW health services	0.0	8.5	91.5	8.0
Better health care-seeking behaviour of individuals and communities	3.2	24.7	72.1	7.3
Health-promoting behaviours in homes and communities	1.1	24.7	74.2	7.5
Improved patient satisfaction	0.0	26.6	73.4	7.4
Decrease in preventable mortality rates	5.3	12.8	81.9	7.7
Improved family planning	8.6	33.3	58.1	6.8
Increased equity	5.3	27.4	67.4	7.1
Improved cost savings by patients	4.4	34.8	60.9	6.7
Decreased morbidity rates	3.2	24.2	72.6	7.3
Decrease in levels of discrimination	6.5	44.6	48.9	6.4

### Perceived acceptability and feasibility ratings by stakeholders

**Fig. 5:**
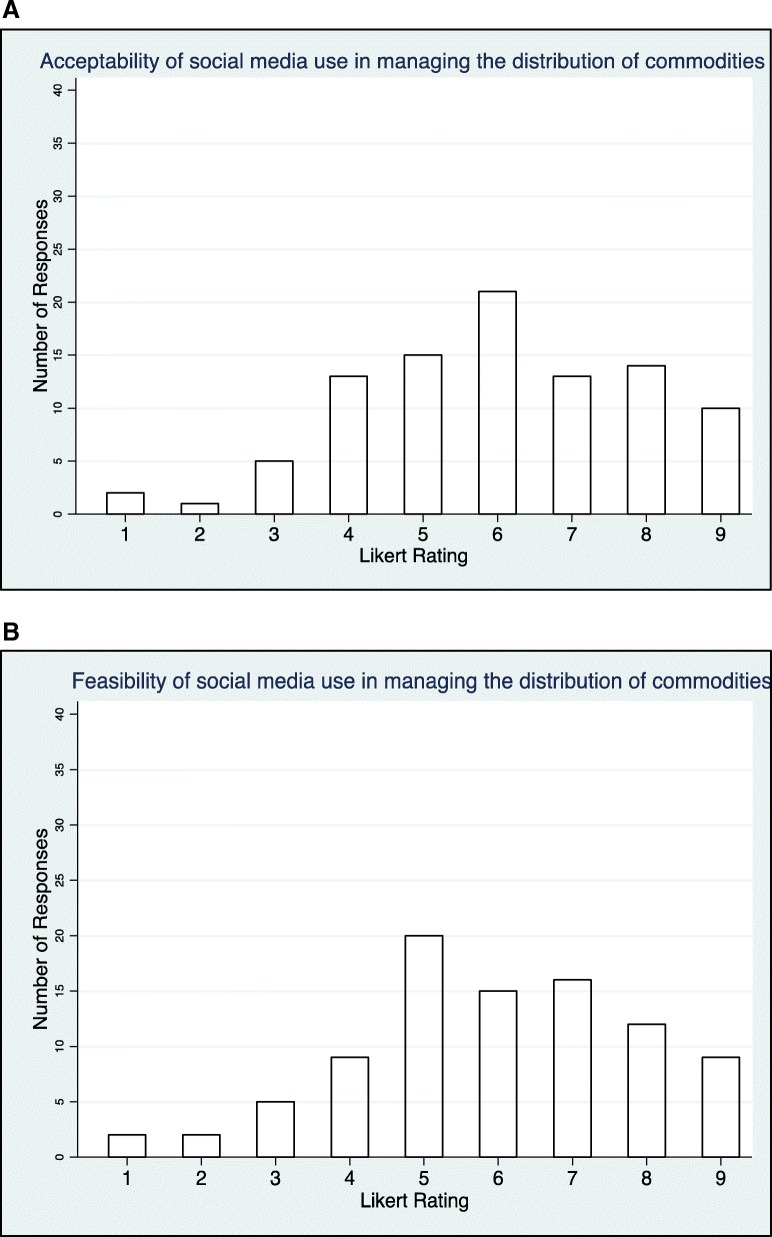
**a** The spread of the number of respondents and the value of the Likert ratings accorded to the acceptability of the policy option of CHW social media use in managing the distribution of commodities and supplies (*n* = 96). **b** The spread of the number of respondents and the value of the Likert ratings accorded to the feasibility of the policy option of CHW social media use in managing the distribution of commodities and supplies (*n* = 96)

**Fig. 6:**
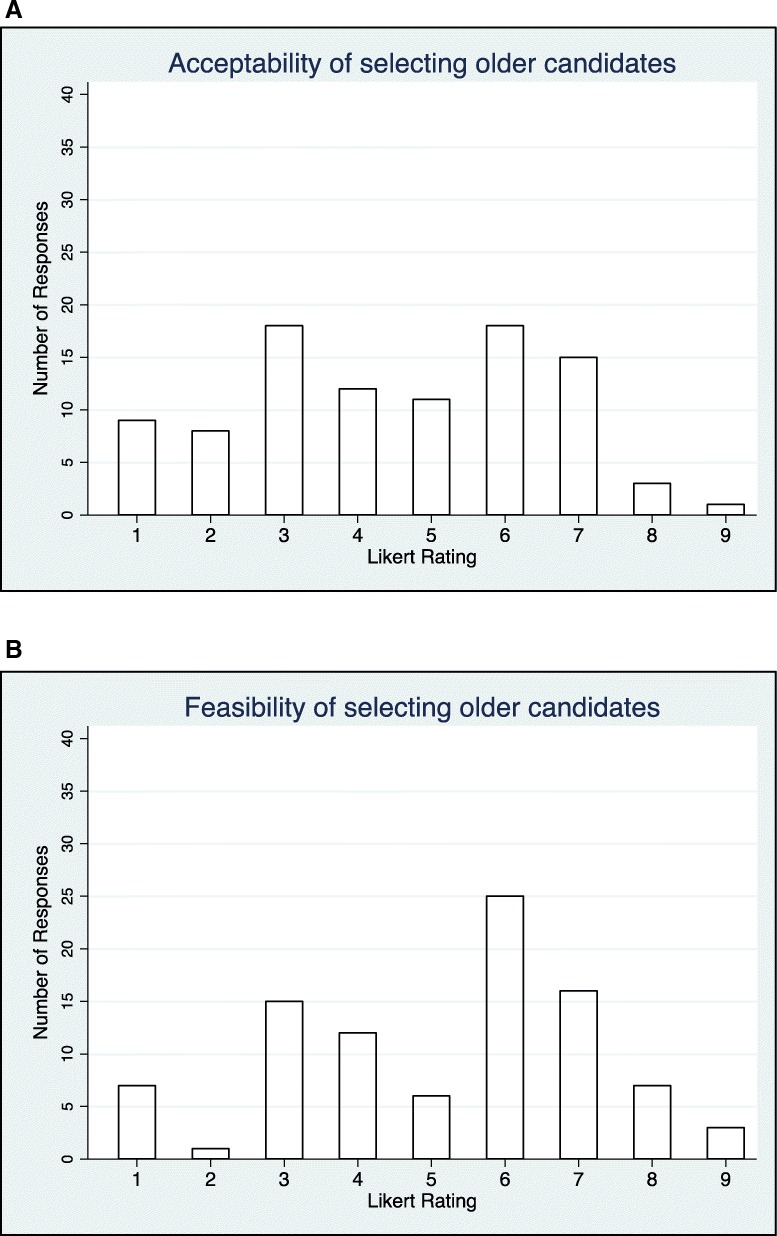
**a** The spread of the number of respondents and the value of the Likert ratings accorded to the acceptability of the policy option to select older CHW candidates. (*n* = 96). **b** The spread of the number of respondents and the value of the Likert ratings accorded to the feasibility of the policy option to select older CHW candidates (*n* = 96)

Table 2 summarizes the perceived ratings of the feasibility and acceptability of the policy options considered for the CHW guideline. Based on the percentage of participants responding to the acceptability of the policy options, *CHWs collecting and submitting routine data* was considered the most ‘definitely acceptable’ at 96%. Following in the second place and third places for ‘definitely acceptable’, policy options are *proactive community mobilization by CHWs* (93%) and *community engagement strategies* (92%) respectively. For policy options rated as ‘definitely not acceptable’, *selecting older candidates on the basis of age* (37%), *adopting only CHWs who have completed a minimum of secondary education* (29%) and *assessing CHWs by service delivery supervision only* (29%) were judged the highest in descending order. Figure 5a and b and Fig. 6a and b help to illustrate the ‘middle spread’ of the frequency distribution of the policy interventions for which there was the least certainty regarding acceptability or feasibility.

**Table 2:** Table comparing the frequency distribution of the acceptability and feasibility of the policy options under consideration

Policy options	Acceptability			Feasibility			*P* value†	Mean score for acceptability (*n* = 95)	Mean score for feasibility (*n* = 92)
	DNA (%)	UA (%)	DA (%)	DNF (%)	UF (%)	DF (%)			
1. Compared to other methods or no assessment at all, how acceptable is the use of this questionnaire to rate the acceptability by stakeholders of implementing CHW policy interventions?	4	41	54	10	33	57	0.24*	6.3	6.2
Selection, education and certification									
2. Using essential and desirable attributes to select CHWs for pre-service training	0	16	84	3	12	85	0.17*	7.3	7.2
(a) Adopting only CHWs who have completed a minimum of secondary education (relative to lower levels of literacy)	29	35	36	29	25	46	0.27	5.2	5.5
(b) Selecting older candidates on the basis of age (relative to random age selection)	37	43	20	25	47	28	0.17	4.5	5.2
(c) Selecting members of the target community (relative to selecting non-members)	5	28	67	9	20	71	0.36	6.9	7.0
3. Training of CHWs for a short period (could range from a number of days to 1 month relative to training for a longer period of 6 months to 3 years)	13	26	62	7	20	74	0.16	6.4	7.0
4. Having standardized educational curricula	8	22	71	9	20	72	0.92	6.8	7.0
(a) Curricula addressing biological /medical (determinants, basic notions of human physiology, pharmacology, and diagnosis and treatment)	22	35	43	19	35	46	0.8	5.6	5.8
(b) Curricula addressing household level preventive behaviours in relation to priority health conditions	1	7	91	1	11	88	0.71*	7.9	7.8
(c) Curricula addressing education about social determinants of health	1	13	86	2	13	85	0.83*	7.6	7.6
(d) Curricula addressing counselling and motivation skills (including communication skills)	1	7	92	1	9	90	0.87*	8.0	7.8
(e) Curricula addressing scope of practice (attitude, when to refer patients, range of tasks, power relationships with the client, personal safety)	1	13	86	1	13	86	1*	7.9	7.8
(f) Curricula should address CHW integration within the wider system (access to resources)	2	14	84	2	18	80	0.78*	7.7	7.5
5. Issuing a formal certification for CHWs who have undergone competency-based pre-service training	3	15	82	2	16	81	0.89*	7.6	7.6
Management and supervision									
6. Strategic supervision support for CHWs	0	9	91	1	13	86	0.34*	8.2	7.8
(a) Coaching of CHWs	0	11	89	4	12	84	0.12*	8.0	7.5
(b) Use of task checklists	1	13	86	1	9	90	0.7*	7.9	7.8
(c) Observation of CHWs at facility	7	21	73	5	20	75	0.93	7.1	7.2
(d) Observation of CHWs at community and facility	2	12	86	2	11	87	0.98*	7.8	7.6
(e) CHWs supervising CHWs	16	32	52	14	28	58	0.76	6.1	6.3
(f) Higher cadre health workers supervising CHWs	3	11	86	2	17	81	0.48*	7.7	7.5
(g) Trained supervisor	3	8	89	0	9	91	0.22*	7.9	7.8
(h) Assessing CHWs by service delivery supervision only	29	40	31	15	27	58	< 0.01	5.2	6.3
(i) Assessing CHWs by service delivery supervision and community feed-back	3	10	87	1	21	78	0.07*	7.6	7.4
7. Rewarding CHWs for their work	1	14	85	3	11	86	0.51*	7.9	7.6
(a) Monetary incentives	5	29	66	13	24	63	0.17	7.2	6.7
(b) Non-monetary incentives	8	19	73	7	19	75	0.95	7.2	7.1
(c) Benchmarking full-time CHW salary to the government minimum wage of the locality	11	31	59	18	29	52	0.31	6.7	6.2
8. CHWs having a career ladder opportunity/ framework within the health and education systems	6	18	76	13	34	53	< 0.01	7.3	6.4
Integration in and support by health system and communities									
9. CHWs having a formal contract within the health system	5	24	71	10	30	60	0.24	7.0	6.7
10. CHWs collecting and submitting data on their routine activities	1	3	96	1	11	88	0.12*	8.0	7.7
11. Community engagement strategies to support practicing CHWs (including village health committees and community health action planning activities)	1	7	92	0	13	87	0.27*	7.9	7.6
12. Proactive community mobilization by CHWs (identifying priority health and social problems, mobilizing local resources, engaging communities in participation of health service organization and delivery)	0	7	93	1	15	84	0.14*	8.0	7.5
13. Providing strategies to ensure adequate availability of commodities and consumable supplies in the context of practicing CHW programmes	1	11	88	1	16	83	0.61*	7.9	7.4
(a) Ensuring inclusion of relevant commodities in the National Pharmaceutical Supply Plan or equivalent national supply chain plan	2	16	82	2	21	76	0.64*	7.9	7.3
(b) Simplified stock management tools and visual job aids for CHWs that accommodate low literacy with minimum data points to facilitate recording of data and re-supply	1	9	90	1	15	84	0.43*	8.0	7.6
(c) Use of mobile phone applications (mHealth) for reporting stock and other data	0	20	80	3	31	66	0.04*	7.4	7.0
(d) Co-ordination, supervision and standardization of resupply procedures, checklists and incentives	1	11	88	1	20	79	0.22*	7.8	7.3
(e) Products specifically designed for use by CHWs (presentation, strength, form and packaging)	4	19	77	5	25	69	0.53*	7.3	7.0
(f) Use of social media to manage commodity distribution	9	52	39	10	49	41	0.89	6.0	6.0

*DNA* definitely not acceptable, *UA* uncertain whether acceptable or not, *DA* definitely acceptable, *DNF* definitely not feasible, *UF* uncertain whether feasible or not, *DF* definitely feasible

*These statistics should be interpreted with caution as at least one cell contained less than 5 observations

†Chi-square comparing acceptability distribution with feasibility *P* value

Regarding the feasibility of the policy options, *having a trained supervisor* was judged to be the most ‘definitely feasible’. Coming next were *use of task checklists* (91%) and *CHW curricula addressing counselling and motivational skills* (91%). For ‘uncertain whether feasible or not’, *use of social media to manage redistribution* was rated the highest with 49% of the total responders. *Selecting older candidates on the basis of age* was rated second with 47% while *curricula addressing biological/medical competencies* came in third with 35% of responders. For policy options that were judged to be ‘definitely not feasible’, *adopting only CHWs who have completed a minimum of secondary education* was rated highest with 29% of respondents. *Selecting older candidates on the basis of age* was rated second with 25% and *curricula addressing biological/medical contents* was third with 19%.

### Qualitative findings

Comments were provided by the survey participants to provide qualitative information related to the choice of their outcomes and policy option ratings and to provide CHW experiences related mostly to specific practice settings. It was generally noted that the monitoring and evaluation of outcomes should be context-specific and context-driven.

On selection, education and training, and certification of CHWs, participants mentioned that the development of standardized educational curricula should be based on community needs and appropriate information that is understandable by the CHWs. It was also noted that CHW curricula should be tailored to local priorities and not include overly complex biological and medical issues. The inclusion of skills necessary for solving community problems is important. On the possession of educational qualifications, one responder commented, ‘In many settings, mandatory educational criteria severely drains the pipeline of otherwise qualified CHWs, and this is particularly acute when gender inequalities are considered’. As such, the responder suggested that CHWs should not be selected based on certified qualifications but on skills possessed.

On management and supervision, respondents thought that recruiting from communities should be strongly preferred and training be continued throughout employment. CHWs based in the community should be supervised within that community. On remuneration, one stakeholder mentioned that ‘the debate should centre around the best way to structure salaries and incentives, not whether they should exist at all’. It was also mentioned that ‘career advancement opportunities increase [the] quality of candidates recruited and the performance of CHWs while delivering services’.

Other relevant submissions on health systems support include attestations to the importance of including CHWs in developing tools such as simplified checklists, stock management tools and visual job aids that accommodate low literacy use in recording of data and the supply of commodities. It was remarked that such moves would facilitate community engagement and better use of the tools. Data collection as part of larger health system accountability and learning systems was also noted to be important.

## Discussion

The results of the study show that stakeholders rate the policy options to be generally acceptable and feasible. Participants deemed most of the outcomes of interest to be close to the ‘critical’ end of the scale with the highest-ranking outcomes being *quality of CHW health services*, *health services coverage* and *access to care for patients*. Participants also judged most of the policy options under consideration as ‘definitely acceptable’ and ‘definitely feasible’. They were uncertain regarding the acceptability or feasibility of few options, particularly the *selection of CHWs on the basis of age* and *the selection of only those who completed a minimum secondary level of education*. *Adopting only CHWs who have completed a minimum of secondary education* and the *assessment of CHWs by service delivery supervision only* were rated the most unacceptable (29%) for both policy options. It also follows that both policy options received the least percentage score for whether they are ‘definitely acceptable’ at 36% and 31% respectively.

Comparing the acceptability and feasibility of the policy options using the chi-square test, two are found to have statistically significant differences when *p* value < 0.05. These are *assessing CHWs by service delivery supervision only* and *CHWs having a career ladder opportunity* within the health and education systems. In the former, respondents found it more ‘definitely feasible’ (58%) than ‘definitely acceptable’ (31%). For the latter policy option, respondents judged it more ‘definitely acceptable’ (76%) than ‘definitely feasible’ (53%). Stakeholders and implementers may want to examine more contextual factors to determine setting-specific approaches regarding these policy options. For example, creating *career ladder opportunities for CHWs* may be acceptable but judging the required level of effort and resources needed to implement it will be important in this context. Similarly, though *assessing CHWs by service delivery supervision only* may be judged as feasible, the level of acceptance may require more caution and sensitivity if considered for application.

These study findings are important as they provide a useful signal from possible users on the relevance of the policy options, in addition to evidence obtained from systematic reviews in developing the guidelines. A systematic review of existing reviews on CHWs by Scott et al. documents the various ways in which CHWs have been deployed and the levers that helped ensure their success [20]. Findings of this study reveal that international/multilateral stakeholders were more represented (43%) in the survey compared to stakeholders representing governmental organizations (19%). Which stakeholders to engage and how to engage them will vary between settings. Integrating CHWs into health systems, however, is likely to ensure the sustainability of CHW programmes, and it is important for programme planners and decision makers to consider their approach according to health priorities and management and resource needs. An interesting observation narrated through the qualitative feedback highlights the importance of ensuring gender equality in selecting CHWs. In some contexts where female education is noted to be generally lower than that of males, it may be important to consider (while ensuring that quality and capacity are not undermined) that educational criteria or entry qualification standards for CHWs do not disadvantage women from selection in the first place.

### Strengths and limitations of the study

The completion rate among those who responded to the questionnaire was above 95%. A limitation to the study was an inability to calculate the response rate of the questionnaire. This was because the exact number of stakeholders involved with managing CHWs programmes (as the primary targets) could not be assessed from our sampling pool. Also, CHWs themselves constituted a minority among the survey responders. This was accounted for during the guideline development process by ensuring that at least two CHWs were part of the GDG. The use of online surveys and respondent anonymity precluded direct follow-up for more information. There was also a lack of participation from stakeholders from the Eastern Mediterranean region.

This paper on perceptions of policy options to support the integration of community health workers is guided by best practice approaches to support systematic evidence analysis for policy guideline development on acceptability and feasibility of policy options for end-users. While perception surveys have been conducted for CHWs, guideline perception surveys are not widespread. No peer-reviewed literature was found to support an established procedure for conducting such studies. Accordingly, we have aligned the purpose of this paper with that earlier conducted by Darzi et al. [18] though the survey questionnaire and the analytic methodology were varied slightly to suit the context of this paper. While this study may be described as a pre-intervention study, related post intervention studies [21, 22] also have been conducted. For example, Buchner DL et al. conducted a 2014 study in Uganda on stakeholders’ perceptions of Integrated Community Case Management (iCCM) by community health workers. The study, which examined the impact of trained CHWs offering health services in a county district, concluded that CHWs may improve access to health care and that CHWs provision of health services was acceptable to families in their setting.

## Conclusion

The integration of CHWs in health systems is vital for them to thrive in countries. The results of the acceptability and feasibility of most of the described policy options above (though interpreted with caution due to the study limitations) indicate the prospect and willingness of stakeholders to support the integration of CHWs in health systems of countries.

### Implications for practice

National health policies, strategies and plans are more likely to be implemented effectively if their negotiation and development is inclusive of all stakeholders in the health and interrelated sectors [23]. The purpose of this survey was to understand and assess the perception of CHW stakeholders (particularly implementers of CHW programmes) to ensure that guideline and policy formulation incorporate their opinions and integrate practical ideas and solutions, in addition to referencing scientific evidence obtained from systematic reviews.

Considerable evidence-based resources to support CHW programmes are available in the public domain. Synergistic stakeholder partnerships are vital to utilize these resources effectively for CHW capacity building [24–27]. A four-country qualitative study conducted by De Neve, Jan-Walter et al. in South Africa provided five policy recommendations to harmonize CHW programmes in order to strengthen the role of CHWs in HIV service delivery [28]. The study revealed that stakeholders were generally in support of harmonizing CHW programmes. Among the key facilitators to harmonization were the presence of a large existing national CHW programme and the recognition of non-governmental organization CHW programmes, use of common incentives and training processes for CHWs and the existence of organizational structures dedicated to community health initiatives.

### Implications for research

The outcome measures and the acceptability and feasibility of the policy options described in earlier sections were all deemed to be important or critical and mostly acceptable/feasible respectively. Interpreting the Likert rating, no outcome measure was rated as ‘not important’, nor were any interventions deemed to be ‘definitely not acceptable’ or ‘definitely not feasible’. Combining our interpretation of the quantitative and qualitative data, we presume that the guidelines contents are relevant to interested stakeholders, paving the way for increased acceptance, political will and enthusiasm to implement the recommendations of the guidelines in countries. Further research should examine acceptability and feasibility considerations in the context of the application and implementation of the guideline recommendations. Such information could provide evidence for updating the CHW guideline and developing optimal implementation strategies.

## Additional files

Additional file 1: Survey questionnaire for acceptability and feasibility of CHW guideline policy options. (PDF 1310 kb)

## Abbreviations

CHW: Community health worker
ERC: Ethics Review Committee
ERG: External Review Group
GDG: Guideline Development Group
GRADE: Grading of Recommendations Assessment, Development and Evaluations
GRC: Guidelines Review Committee
HIFA: Health Information for All
iCCM: Integrated Community Case Management
ILO: International Labour Organization
NGO: Non-governmental organization
PHC: Primary health care
PICO: Population, Intervention, Comparison, Outcome
SDG: Sustainable Development Goal
UHC: Universal health coverage
WHO: World Health Organization
