# Supplementary material for: Stakeholders’ perceptions of policy options to support the integration of community health workers in health systems
Source: Hum Resour Health. 2019 Feb 18;17:13. doi: 10.1186/s12960-019-0348-6 (PMC6379925; doi:10.1186/s12960-019-0348-6)
Supplement: Supplementary file 1 — Survey questionnaire for acceptability and feasibility of CHW guideline policy options. (PDF 1310 kb) [file 12960_2019_348_MOESM1_ESM.pdf]

World Health Organization

Stakeholders’ perception survey on the implementation of the WHO guidelines on health policy and system support to optimize community health worker programmes

Policy guidelines on health policy and system support to optimize community health worker programmes

Introduction

The World Health Organization (WHO) is developing policy guidelines on health policy and system support to optimize community health worker programmes. We are conducting the present survey to better understand the views of stakeholders in relation to the feasibility and acceptability of some of the interventions and recommendations under consideration, and the values assigned to the outcomes of the interventions.

Note to participants: This survey asks for your perception about the feasibility and acceptability of the interventions and your assessment of how important the outcome measures are to ensure successful implementation. The information obtained will be analysed and primarily used to support the ongoing development of the guidelines on health systems and policy support for community health workers. Responses provided are anonymous and remain confidential; only aggregate findings will be presented and/ or published by WHO. Participation in the survey is voluntary; you have the right to refuse to respond to any or all questions, but we kindly request that you answer as many questions as possible in order to help us to have relevant and helpful information. We will consider your consent is given when you choose to take the survey and “pressing NEXT” as recognition of your voluntary participation. Completion of the questionnaire should take approximately 20 minutes and you will be able to save your answers as you go along, or return to them at a later time. Please complete the ratings from you own perspective, taking into account your specific/local context. You can find descriptions of the outcome measures in the accompanying glossary.

You may also choose to add explanations to your ratings or offer other comments in the open questions at the end.

Should you encounter any questions on which you would like further clarification or guidance, please feel free to contact ajueboro@who.int with your questions. You can find more information regarding the guideline development process at www.who.int/hrh/community/en/

Thank you for completing this survey!

* 1. Age

- 18 to 30
- 31 to 50
- 51 to 64
- > 64

2. Gender

- Female
- Male

* 3. Institution

- Non-governmental organization (NGO) or community Based organization (CBO)
- International/ multilateral organization (NGO)
- Governmental organization
- Academic/ research institution
- Private for profit organization
- Other

4. If you answered other to the question 3 above, please specify

* 5. Highest attained education degree

- Primary school certificate
- Secondary school certificate or diploma
- Bachelor's degree (e.g. BSc)
- Master's degree (e.g. MSc, MA)
- Doctoral degree (MD, PhD or equivalent)
- None of the above

* 6. WHO Region

- ○ Africa
- ○ South and South East Asia
- ○ Americas
- ○ Europe
- ○ The Eastern Mediterranean
- ○ The Western Pacific

* 7. Please specify your Country

[ ]

* 8. Occupation

- [ ] Community health worker
- [ ] Health Professional or health worker (other than community health worker) involved in the direct delivery of health services :
- [ ] Health policy maker/ health planner
- [ ] Health services manager and/ or administrator
- [ ] Other

9. If you answered other to question 8 above, please specify

[ ]

* 10. Level of responsibility

- [ ] District (within a country)
- [ ] National (at the country level)
- [ ] Regional (at a level broader than national)
- [ ] International
- [ ] Not applicable

11. Please rate on the scale of 1-9, how important you think the following results measures are for assessing the outputs, outcomes and impact of the CHW guideline recommendations

	Not important (1)	Not important (2)	Not important (3)	Important (4)	Important (5)	Important (6)	Critical (7)	Critical (8)	Critical (9)
Increased CHW **motivation**	○	○	○	○	○	○	○	○	○
Improved CHW **morale**	○	○	○	○	○	○	○	○	○
Decreased CHW **absenteeism**	○	○	○	○	○	○	○	○	○
Increased CHW **productivity**	○	○	○	○	○	○	○	○	○
Improved CHW **responsiveness**	○	○	○	○	○	○	○	○	○
Decrease in CHW **attrition** rates	○	○	○	○	○	○	○	○	○
Improved CHW **competencies**	○	○	○	○	○	○	○	○	○
Increased **access** to care for patients	○	○	○	○	○	○	○	○	○
Increased health services **coverage**	○	○	○	○	○	○	○	○	○
Improved **quality** of CHW health services	○	○	○	○	○	○	○	○	○
Better **health care-seeking behaviour** of individuals and communities	○	○	○	○	○	○	○	○	○
**Health-promoting behaviours** in homes and communities	○	○	○	○	○	○	○	○	○
Improved **patient satisfaction**	○	○	○	○	○	○	○	○	○
Decrease in preventable **mortality** rates	○	○	○	○	○	○	○	○	○
Improved **family planning**	○	○	○	○	○	○	○	○	○
Increased **equity**	○	○	○	○	○	○	○	○	○
Improved **cost savings** by patients	○	○	○	○	○	○	○	○	○
Decreased **morbidity** rates	○	○	○	○	○	○	○	○	○
Decrease in levels of **discrimination**	○	○	○	○	○	○	○	○	○

12. Please rate on a scale of 1-9, how**acceptable** for implementation you think the following CHW interventions are

	Definitely not acceptable (1)	Definitely not acceptable (2)	Definitely not acceptable (3)	Uncertain whether acceptable or not (4)	Uncertain whether acceptable or not (5)	Uncertain whether acceptable or not (6)	Definitely acceptable (7)	Definitely acceptable (8)	Definitely acceptable (9)
1) Compared to other methods or no assessment at all, how acceptable is the use of this questionnaire to rate the acceptability by stakeholders of implementing CHW policy interventions?									
2) Using essential and desirable attributes to select CHWs for pre-service training									
a) Adopting only CHWs who have completed a minimum of secondary education (relative to lower levels of literacy)									
b) Selecting older candidates on the basis of age (relative to random age selection)									
c) Selecting members of the target community over non-members									
3) Training of CHWs for a short period (relative to task and could range from a number of days to one month) as against training for a longer period (6 months to 3 years)									
4) CHWs should have standardized educational curricula									

	Definitely not acceptable (1)	Definitely not acceptable (2)	Definitely not acceptable (3)	Uncertain whether acceptable or not (4)	Uncertain whether acceptable or not (5)	Uncertain whether acceptable or not (6)	Definitely acceptable (7)	Definitely acceptable (8)	Definitely acceptable (9)
a) Curricula should address biological /medical (determinants, basic notions of human physiology, pharmacology, and diagnosis and treatment)	○	○	○	○	○	○	○	○	○
b) Curricula should address household level preventative behaviours in relation to priority health conditions	○	○	○	○	○	○	○	○	○
c) Curricula should address education about social determinants of health	○	○	○	○	○	○	○	○	○
d) Curricula should address counselling and motivation skills (including communication skills)	○	○	○	○	○	○	○	○	○
e) Curricula should address scope of practice (attitude, when to refer patients, range of tasks, power relationships with the client, personal safety)	○	○	○	○	○	○	○	○	○
f) Curricula should address CHW integration within the wider system (access to resources)	○	○	○	○	○	○	○	○	○
5) Issuing a formal certification for CHWs who have undergone competency-based pre-service training	○	○	○	○	○	○	○	○	○
6) Strategic supervision support for CHWs	○	○	○	○	○	○	○	○	○

	Definitely not acceptable (1)	Definitely not acceptable (2)	Definitely not acceptable (3)	Uncertain whether acceptable or not (4)	Uncertain whether acceptable or not (5)	Uncertain whether acceptable or not (6)	Definitely acceptable (7)	Definitely acceptable (8)	Definitely acceptable (9)
a) Coaching	○	○	○	○	○	○	○	○	○
b) Use of task checklists	○	○	○	○	○	○	○	○	○
c) Observation of CHWs at facility	○	○	○	○	○	○	○	○	○
d) Observation of CHWs at community and facility	○	○	○	○	○	○	○	○	○
e) CHWs supervising CHWs	○	○	○	○	○	○	○	○	○
f) CHWs supervised by higher cadre health workers	○	○	○	○	○	○	○	○	○
g) Trained supervisor	○	○	○	○	○	○	○	○	○
h) Assessing CHWs by service delivery supervision only	○	○	○	○	○	○	○	○	○
i) Assessing CHWs by service delivery supervision and community feed-back	○	○	○	○	○	○	○	○	○
7) Rewarding CHWs for their work	○	○	○	○	○	○	○	○	○
a) Monetary payment	○	○	○	○	○	○	○	○	○
b) Non-monetary incentives	○	○	○	○	○	○	○	○	○
c) Benchmarking full-time CHW salary to the government minimum wage of the locality	○	○	○	○	○	○	○	○	○
8) CHWs having a career ladder opportunity/ framework within the health and education systems	○	○	○	○	○	○	○	○	○

	Definitely not acceptable (1)	Definitely not acceptable (2)	Definitely not acceptable (3)	Uncertain whether acceptable or not (4)	Uncertain whether acceptable or not (5)	Uncertain whether acceptable or not (6)	Definitely acceptable (7)	Definitely acceptable (8)	Definitely acceptable (9)
9) CHWs having a formal contract within the health system	○	○	○	○	○	○	○	○	○
10) CHWs collecting and submitting data on their routine activities	○	○	○	○	○	○	○	○	○
11) Community engagement strategies to support practicing CHWs (including village committees and community health action planning activities)	○	○	○	○	○	○	○	○	○
12) Proactive community mobilization by CHWs (identifying priority health and social problems, mobilizing local resources, engaging communities in participation of health service organization and delivery)	○	○	○	○	○	○	○	○	○
13) Providing strategies to ensure adequate availability of commodities and consumable supplies in the context of practicing CHW programmes	○	○	○	○	○	○	○	○	○
a) Ensuring inclusion of relevant commodities in the National Pharmaceutical Supply Plan or equivalent national supply chain plan	○	○	○	○	○	○	○	○	○

	Definitely not acceptable (1)	Definitely not acceptable (2)	Definitely not acceptable (3)	Uncertain whether acceptable or not (4)	Uncertain whether acceptable or not (5)	Uncertain whether acceptable or not (6)	Definitely acceptable (7)	Definitely acceptable (8)	Definitely acceptable (9)
b) Simplified stock management tools and visual job aids for CHWs that accommodate low literacy with minimum data points to facilitate recording of data and re-supply	○	○	○	○	○	○	○	○	○
c) Use of mobile phone applications (mHealth) for reporting stock and other data	○	○	○	○	○	○	○	○	○
d) Co-ordination, supervision and standardization of resupply procedures, checklists and incentives	○	○	○	○	○	○	○	○	○
e) Products specifically designed for use by CHWs (presentation, strength, form and packaging)	○	○	○	○	○	○	○	○	○
f) Use of social media to manage redistribution	○	○	○	○	○	○	○	○	○

13. Please rate on a scale of 1-9, how**feasible** for implementation you think the following CHW interventions are.

	Definitely not feasible (1)	Definitely not feasible (2)	Definitely not feasible (3)	Uncertain whether feasible or not (4)	Uncertain whether feasible or not (5)	Uncertain whether feasible or not (6)	Definitely feasible (7)	Definitely feasible (8)	Definitely feasible (9)
1) Compared to other methods or no assessment at all, how feasible is the use of this questionnaire to rate the feasibility of implementing CHW policy interventions?	○	○	○	○	○	○	○	○	○
2) Using essential and desirable attributes to select CHWs for pre-service training	○	○	○	○	○	○	○	○	○

	Definitely not feasible (1)	Definitely not feasible (2)	Definitely not feasible (3)	Uncertain whether feasible or not (4)	Uncertain whether feasible or not (5)	Uncertain whether feasible or not (6)	Definitely feasible (7)	Definitely feasible (8)	Definitely feasible (9)
a) Adopting only CHWs who have completed a minimum of secondary education (relative to lower levels of literacy)	○	○	○	○	○	○	○	○	○
b) Selecting older candidates on the basis of age (relative to random age selection)	○	○	○	○	○	○	○	○	○
c) Selecting members of the target community over non-members	○	○	○	○	○	○	○	○	○
3) Training of CHWs for a short period (relative to task and could range from a number of days to one month) as against training for a longer period (6 months to 3 years)	○	○	○	○	○	○	○	○	○
4) CHWs should have standardized educational curricula	○	○	○	○	○	○	○	○	○
a) Curricula should address biological /medical (determinants, basic notions of human physiology, pharmacology, and diagnosis and treatment)	○	○	○	○	○	○	○	○	○
b) Curricula should address household level preventative behaviours in relation to priority health conditions	○	○	○	○	○	○	○	○	○
c) Curricula should address education about social determinants of health	○	○	○	○	○	○	○	○	○
d) Curricula should address counselling and motivation skills (including communication skills)	○	○	○	○	○	○	○	○	○

	Definitely not feasible (1)	Definitely not feasible (2)	Definitely not feasible (3)	Uncertain whether feasible or not (4)	Uncertain whether feasible or not (5)	Uncertain whether feasible or not (6)	Definitely feasible (7)	Definitely feasible (8)	Definitely feasible (9)
e) Curricula should address scope of practice (attitude, when to refer patients, range of tasks, power relationships with the client, personal safety)	○	○	○	○	○	○	○	○	○
f) Curricula should address CHW integration within the wider system (access to resources)	○	○	○	○	○	○	○	○	○
5) Issuing a formal certification for CHWs who have undergone competency-based pre-service training	○	○	○	○	○	○	○	○	○
6) Strategic supervision support for CHWs	○	○	○	○	○	○	○	○	○
a) Coaching	○	○	○	○	○	○	○	○	○
b) Use of task checklists	○	○	○	○	○	○	○	○	○
c) Observation of CHWs at facility	○	○	○	○	○	○	○	○	○
d) Observation of CHWs at community and facility	○	○	○	○	○	○	○	○	○
e) CHWs supervising CHWs	○	○	○	○	○	○	○	○	○
f) CHWs supervised by higher cadre health workers	○	○	○	○	○	○	○	○	○
g) Trained supervisor	○	○	○	○	○	○	○	○	○
h) Assessing CHWs by service delivery supervision only	○	○	○	○	○	○	○	○	○
i) Assessing CHWs by service delivery supervision and community feed-back	○	○	○	○	○	○	○	○	○
7) Rewarding CHWs for their work	○	○	○	○	○	○	○	○	○
a) Monetary payment	○	○	○	○	○	○	○	○	○
b) Non-monetary incentives	○	○	○	○	○	○	○	○	○

	Definitely not feasible (1)	Definitely not feasible (2)	Definitely not feasible (3)	Uncertain whether feasible or not (4)	Uncertain whether feasible or not (5)	Uncertain whether feasible or not (6)	Definitely feasible (7)	Definitely feasible (8)	Definitely feasible (9)
c) Benchmarking full-time CHW salary to the government minimum wage of the locality	○	○	○	○	○	○	○	○	○
8) CHWs having a career ladder opportunity/ framework within the health and education systems	○	○	○	○	○	○	○	○	○
9) CHWs having a formal contract within the health system	○	○	○	○	○	○	○	○	○
10) CHWs collecting and submitting data on their routine activities	○	○	○	○	○	○	○	○	○
11) Community engagement strategies to support practicing CHWs (including village committees and community health action planning activities)	○	○	○	○	○	○	○	○	○
12) Proactive community mobilization by CHWs (identifying priority health and social problems, mobilizing local resources, engaging communities in participation of health service organization and delivery)	○	○	○	○	○	○	○	○	○
13) Providing strategies to ensure adequate availability of commodities and consumable supplies in the context of practicing CHW programmes	○	○	○	○	○	○	○	○	○
a) Ensuring inclusion of relevant commodities in the National Pharmaceutical Supply Plan or equivalent national supply chain plan	○	○	○	○	○	○	○	○	○

	Definitely not feasible (1)	Definitely not feasible (2)	Definitely not feasible (3)	Uncertain whether feasible or not (4)	Uncertain whether feasible or not (5)	Uncertain whether feasible or not (6)	Definitely feasible (7)	Definitely feasible (8)	Definitely feasible (9)
b) Simplified stock management tools and visual job aids for CHWs that accommodate low literacy with minimum data points to facilitate recording of data and re-supply									
c) Use of mobile phone applications (mHealth) for reporting stock and other data									
d) Co-ordination, supervision and standardization of resupply procedures, checklists and incentives									
e) Products specifically designed for use by CHWs (presentation, strength, form and packaging)									
f) Use of social media to manage redistribution									

14. Where you would like to clarify, please provide an explanation for chosen answers to any of the questions above. (Please indicate the specific number of the question before providing your answer)

15. Other comments: (250 characters max.)

Glossary

Terms and Definition

Knowledge: Degree to which the CHW has the theoretical or practical understanding of the function and tasks assigned to him/her

Competencies: Degree to which the CHW has the skills necessary to carry out the tasks assigned to him/her

Motivation: An individual's degree of willingness to exert and maintain effort on assigned tasks

Morale: The mental and emotional condition (as of enthusiasm, confidence, etc.) of an individual CHW with regard to the function or tasks at hand

Satisfaction: Degree to which CHWs derive personal satisfaction from serving the community, providing good quality services

Absenteeism: Rate at which those CHWs who are supposed to be delivering services habitually fail to appear to carry out their tasks

Service delivery: Quantity and quality of promotional, preventive, and curative services CHWs provide to community members

Responsiveness: The degree to which an individual CHW responds to the needs of an individual client or group within a reasonable time period

Productivity: A CHW's total output per unit of total input

Attrition: The rate at which practicing CHWS resign, retire, or abandon their positions over time

Advancement: The rate at which CHWs are advancing in their skills, competencies, formal responsibilities, and formal status within the community and the formal health system over time

Access: Client's physical and social access to essential services delivered by CHWs

Coverage: The coverage of selected health services among the population served by CHWs

Quality: The quality of services rendered by CHWs

Health care-seeking behaviour: Client in need of essential services and with access to CHWs is routinely seeking and using promotional, preventive and/or curative services CHWs offer

Health-promoting behaviour in home: Client has adopted health-promoting behaviours in the home as a result of contact with CHWs

Satisfaction: Client's reported degree of satisfaction with the services rendered by CHWs

Cost savings: Money not spent by client that he/she otherwise would have spent (on transportation and other items) in the absence of a CHW

Patient health:
Change in client's state of illness, wellness, survival

Morbidity: Change in the prevalence of serious illness in the population served by CHWs

Mortality: Change in the level of mortality in the population served by CHWs

Fertility rate: The ratio of live births in a CHW-served area to the population of that area expressed per 1,000 population per year

Equity: Degree to which access, coverage, or morbidity/mortality levels vary among different socio-economic or socially defined sub-groups in the population served by CHWs

Source: Adapted from Naimoli et al. 2014. Outcome framework of CHW programmes
